# Beta-catenin represses protein kinase D1 gene expression by non-canonical pathway through MYC/MAX transcription complex in prostate cancer

**DOI:** 10.18632/oncotarget.20229

**Published:** 2017-08-12

**Authors:** Bita Nickkholgh, Sivanandane Sittadjody, Michael B. Rothberg, Xiaolan Fang, Kunzhao Li, Jeff W. Chou, Gregory A. Hawkins, K.C. Balaji

**Affiliations:** ^1^ Department of Physiology-Pharmacology, Wake Forest University School of Medicine, Winston-Salem, NC, USA; ^2^ Wake Forest Institute for Regenerative Medicine (WFRM), Wake Forest University School of Medicine, Winston-Salem, NC, USA; ^3^ Department of Urology, Wake Forest Baptist Health, Winston Salem, NC, USA; ^4^ Biology Department, Wake Forest University, Winston-Salem, NC, USA; ^5^ Department of Biostatistical Sciences, Comprehensive Cancer Center, Wake Forest Baptist Health, Winston-Salem, NC, USA; ^6^ Center for Genomics and Personalized Medicine and WFB Comprehensive Cancer Center, Winston-Salem, NC, USA; ^7^ W.G.(Bill) Hefner Veterans Administration Medical Center, Salisbury, NC, USA; ^8^ Department of Cancer Biology, School of Medicine, Wake Forest University, Winston-Salem, NC, USA; ^9^ Current/Present address: Clinical Bioinformatics, New York Genome Center, New York, NY, USA

**Keywords:** prostate cancer, protein kinase D1, beta-catenin, MYC, MAX

## Abstract

Down regulation of Protein Kinase D1 (PrKD1), a novel serine threonine kinase, in prostate, gastric, breast and colon cancers in humans leads to disease progression. While the down regulation of PrKD1 by DNA methylation in gastric cancer and by nuclear beta-catenin in colon cancer has been shown, the regulatory mechanisms in other cancers are unknown. Because we had demonstrated that PrKD1 is the only known kinase to phosphorylate threonine 120 (T120) of beta-catenin in prostate cancer resulting in increased nuclear beta-catenin, we explored the role of beta-catenin in gene regulation of *PrKD1*. An initial CHIP assay identified potential binding sites for beta-catenin in and downstream of *PrKD1* promoter and sequencing confirmed recruitment of beta-catenin to a 166 base pairs sequence upstream of exon 2. Co-transfection studies with *PrKD1*-promoter-reporter suggested that beta-catenin represses *PrKD1* promoter. Efforts to identify transcription factors that mediate the co-repressor effects of beta-catenin identified recruitment of both MYC and its obligate heterodimer MAX to the same binding site as beta-catenin on the *PrKD1* promoter site. Moreover, treatment with MYC inhibitor rescued the co-repressor effect of beta-catenin on *PrKD1* gene expression. Prostate specific knock out of *PrKD1* in transgenic mice demonstrated increased nuclear expression of beta-catenin validating the *in vitro* studies. Functional studies showed that nuclear translocation of beta-catenin as a consequence of PrKD1 down regulation, increases AR transcriptional activity with attendant downstream effects on androgen responsive genes. *In silico* human gene expression analysis confirmed the down regulation of PrKD1 in metastatic prostate cancer correlated inversely with the expression of MAX, but not MYC, and positively with MXD1, a competing heterodimer of MAX, suggesting that the dimerization of MAX with either MYC or MXD1 regulates *PrKD1* gene expression. The study has identified a novel auto-repressive loop that perpetuates PrKD1 down regulation through beta-catenin/MYC/MAX protein complex.

## INTRODUCTION

Protein Kinase D1 (PrKD1) is a novel serine threonine kinase and a founding member of the protein kinase D family. There is increasing evidence that loss of PrKD1 expression contributes to progression of several human cancers including prostate cancer. Several regulatory mechanisms modulate PrKD1 activity such as autoinhibition, phosphorylation, proteolytic degradation, subcellular localization, methylation and various cell-context–dependent PrKD1 activation mechanisms [1-3]. Epigenetic inactivation of *PrKD1* through hypermethylation of the gene promoter has been reported as the mechanism of PrKD1 down regulation in breast and gastric cancer [4, 5]. However, demethylation agents do not consistently increase PrKD1 expression in some other cancers including prostate (unpublished data). Therefore, we explored alternate mechanisms of PrKD1 regulation in prostate cancer.

Transcriptional regulation of genes is under the control of transcription factors, co-activators and co-repressors. Beta-catenin, a known transcriptional co-activator [6], is involved in transcriptional regulation of genes such as the androgen receptor (AR) [7]. We had previously demonstrated that beta-catenin undergoes substrate phosphorylation at Threonine 120 (T120) by PrKD1 [10]. Interestingly, mutation of beta-catenin at T112/T120 increases nuclear translocation suggestive of increased beta-catenin nuclear activity [8]. Similar to the known co-regulatory role of beta-catenin on AR expression, we hypothesized that beta-catenin might also have a regulatory role on PrKD1. Moreover, we explored whether expression and activity of AR could be influenced by beta-catenin mediated PrKD1 regulation. Our study demonstrated that increased nuclear beta-catenin reduces *PrKD1* expression through MYC/MAX transcription factors and thereby perpetuating down regulation of PrKD1 leading to increased nuclear beta-catenin. The study for the first time identifies a novel auto-repressive loop for PrKD1 expression that perpetuates PrKD1 down regulation contributing to disease progression in prostate cancer.

## RESULTS

### Beta-catenin regulates PrKD1 expression by binding to the gene promoter

**Figure 1:**
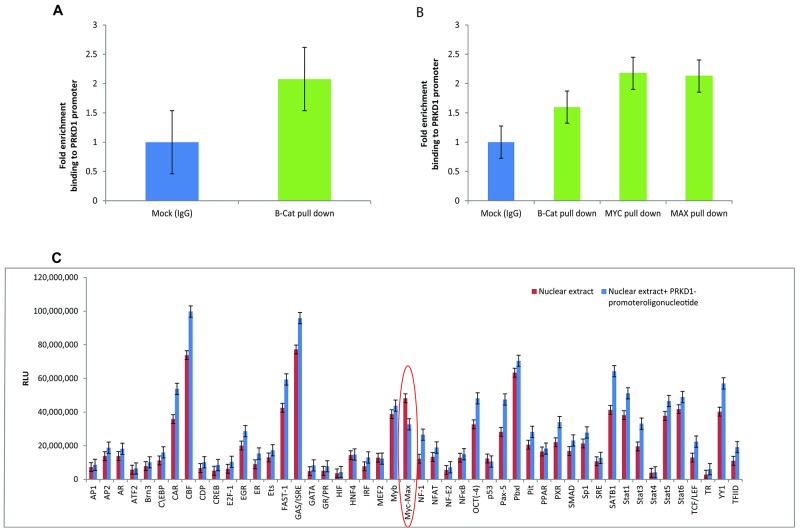
Presence of the beta-catenin and MYC/MAX binding site at the *PrKD1* gene promoter **(A)** Chromatin immunoprecipitation (CHIP) for beta-catenin on LNCaP cells. Cross-linked DNA/protein complex of LNCaP cells was pulled down with beta-catenin antibody or normal IgG antibody (as control). DNA was isolated from the pulled down complex and the enrichment of *PrKD1* promoter sequence in the complex were quantified by qPCR, which shows significantly higher recruitment of beta-catenin to *PrKD1* promoter region compared to IgG control. **(B)** CHIP assay following pull down with MYC and MAX antibodies showed both MYC and MAX are recruited to the same *PrKD1* binding site for beta-catenin. **(C)**Transcription factor (TF) profiling using a 48-TF array. LNCaP nuclear extract pulled down with beta-catenin was mixed with either probe and *PrKD1* promoter sequence or probe alone. The luminescence of the 48-TF array plate quantified and compared between the two samples. The bar graph shows the luminescence intensity for streptavidin. The TFs that regulates *PrKD1* will bind to *PrKD1* sequence and not to the corresponding probes showing lower intensity of bars compared to samples without *PrKD1* sequence. TF binding is considered significant when the intensity for TF in the samples without *PrKD1* is ≥1.5 fold compared to samples containing *PrKD1 sequence* bound to the PrKD1 promoter.

To investigate the effect of beta-catenin on *PrKD1* gene regulation, we performed CHIP assays using high PrKD1 expressing LNCaP prostate cancer cell lysate and beta-catenin antibody. The assay demonstrated recruitment of beta-catenin to the promoter site of *PrKD1* gene as well as other downstream regions in the gene (Figure 1A), which suggests that beta-catenin could be involved in the regulation of PrKD1 expression. To find the exact binding site, we did CHIP sequencing of the DNA pulled down with beta-catenin antibody, which showed that beta-catenin protein complex binds to a 166 bp sequence upstream and near exon 2 (chr14:29899631-29899796). We designed a primer set for this166bp- region and confirmed that beta-catenin is recruited to this area (Figure 1B). Because beta-catenin is a transcriptional co-activator and not a known transcription factor, we sought to identify the transcription factor(s) that mediate the effects of beta-catenin on the *PrKD1* promoter. We performed a TF profiling assay using a promoter array (see Materials and Methods) by adding LNCaP nuclear protein extract (pulled down with beta-catenin) with or without *PrKD1* promoter sequence to 48-TF array plate (the design of the array is shown in Supplementary Figure 1). Results of TF array showed that MYC/MAX complex might be the transcription factor complex that mediates the regulatory effect of beta-catenin on *PrKD1* gene (Figure 1C). As MYC/MAX complex attaches to CAC(G/T)TG E-box sequence on target genes [9], we first checked for the presence of the sequence on the *PrKD1* gene and found at least 14 such palindromic sequences on the gene (Supplementary Table 2). Interestingly, we found one repeat of the sequence on the 166bp sequence that was identified as the same site as beta-catenin was bound to, suggesting MYC/MAX complex could be a reliable TF candidate. We validated the results by performing CHIP assay using MYC and MAX antibodies (Figure 1B). The results showed recruitment of both MYC and MAX to the same *PrKD1* promoter binding site for beta-catenin, which was highly suggestive of MYC/MAX/beta-catenin complex playing a regulatory role in *PrKD1* gene expression.

### Down regulation of PrKD1 increases the nuclear beta-catenin

**Figure 2:**
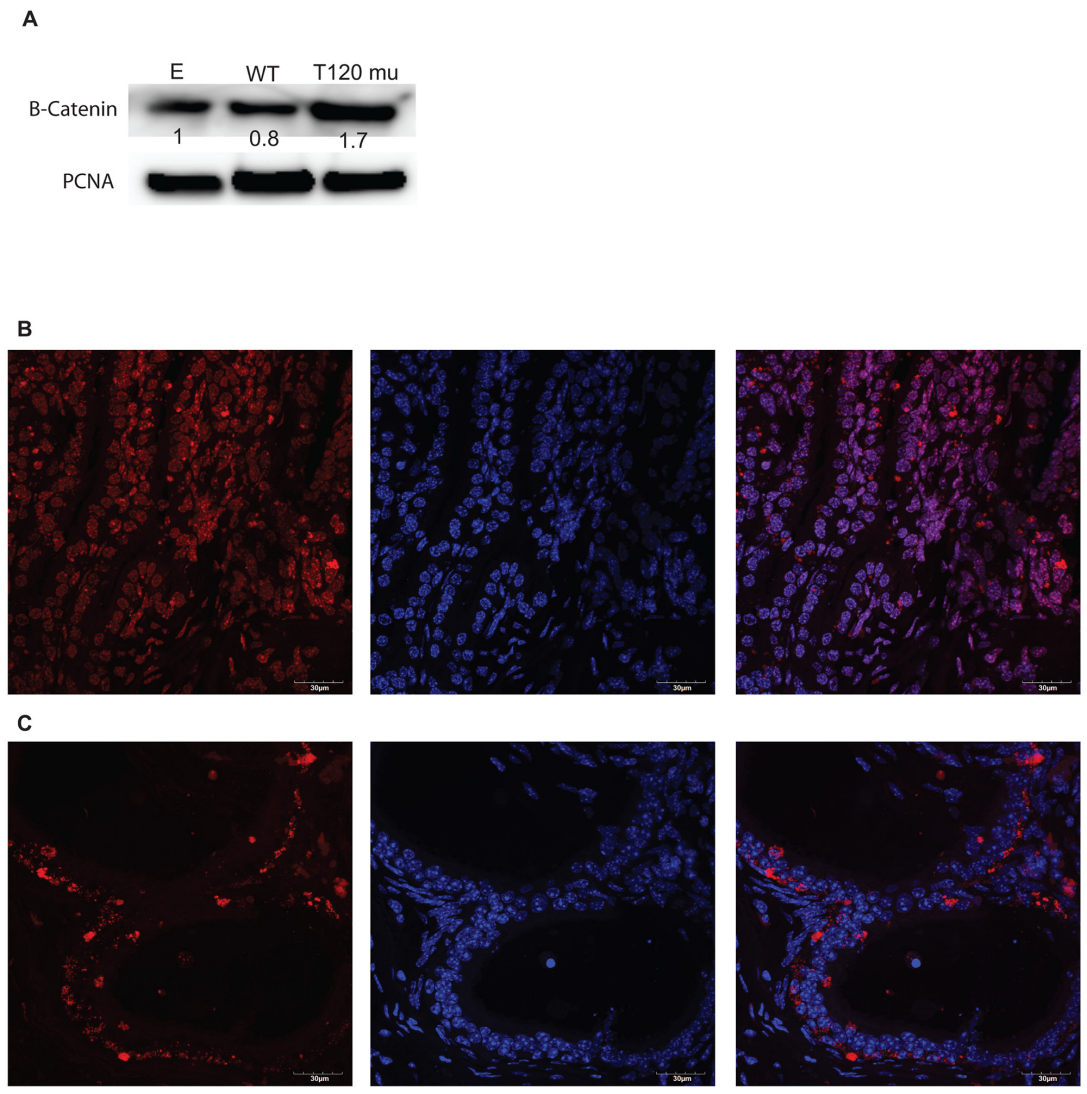
PrKD1 down regulation or representative condition (beta-catenin T120 mutation) increases nuclear beta-catenin *in vitro* and *in vivo* **(A)** The western blot using nuclear fraction of the transfected cells and PCNA as loading control for nuclear protein confirming transfection efficiency. The numbers under beta-catenin bands show the relative density of the bands, calculated as the ratio of each sample density for beta-catenin to the density of the same sample for PCNA. The results for wild type beta-catenin (WT) and T120 mutant (T120 mu) were normalized against empty vector (E). Increased beta-catenin nuclear localization in *PrKD1* knocked out (KO) mice **(B)** compared to litter mate control **(C)**. In B and C panels representative samples of beta-catenin immunofluorescent staining in mouse prostate tissue demonstrating the co- localization of beta-catenin (red, left panel) and nuclear DAPI (blue, middle panel) results in purple color (right panel) under x63 magnification of the confocal microscope. In PrKD1 KO mice (B), beta-catenin shows more nuclear translocation compared to littermate controls (C). In each set of pictures, the left panel: beta-catenin/AF594; middle panel: DAPI nuclear staining and right panel: merged. Scale bar shows 30μM.

As PrKD1 mediates subcellular localization of beta-catenin [8] and down regulation of PrKD1 decreases the phosphorylation of beta-catenin at T120 residue [10], we investigated whether the absence of T120 phosphorylation influences the subcellular localization of beta-catenin. We induced the mutation of T120 (threonine at position 120 on beta-catenin protein) to Isoleucine, which prevents T120 phosphorylation and mimics the effects of PrKD1 down regulation in cells. We stably transfected LNCaP cells (androgen sensitive prostate cancer cell line with high expression of PrKD1 and AR [11]), with T120-mutated beta-catenin, wild type beta-catenin (WT) or empty vector. In addition to confirming the efficacy of transfection through western blot, we also demonstrated that the T120 mutant effects are not due to dominant negative effect on the levels of wild type beta-catenin (Supplementary Figure 2). We observed that unphosphorylated-T120 beta-catenin localized more to the nucleus compared to wild type beta-catenin (WT) (Figure 2A). In order to validate in *vitro* findings using an *in vivo* model, we harvested prostate tissue from prostate specific *PrKD1* C57BL/6J knock out (*PrKD1* KO) mice (n=3) and littermate controls (n=3) [12]. Qualitative comparative analysis by immunofluorescent (IF) confocal microscopy, convincingly demonstrated increased nuclear localization of beta-catenin in the prostate specific *PrKD1* KO mice compared to litter-mate controls (Figure 2B-2C).

### Nuclear beta-catenin has a negative regulatory effect on *PrKD1* promoter

**Figure 3:**
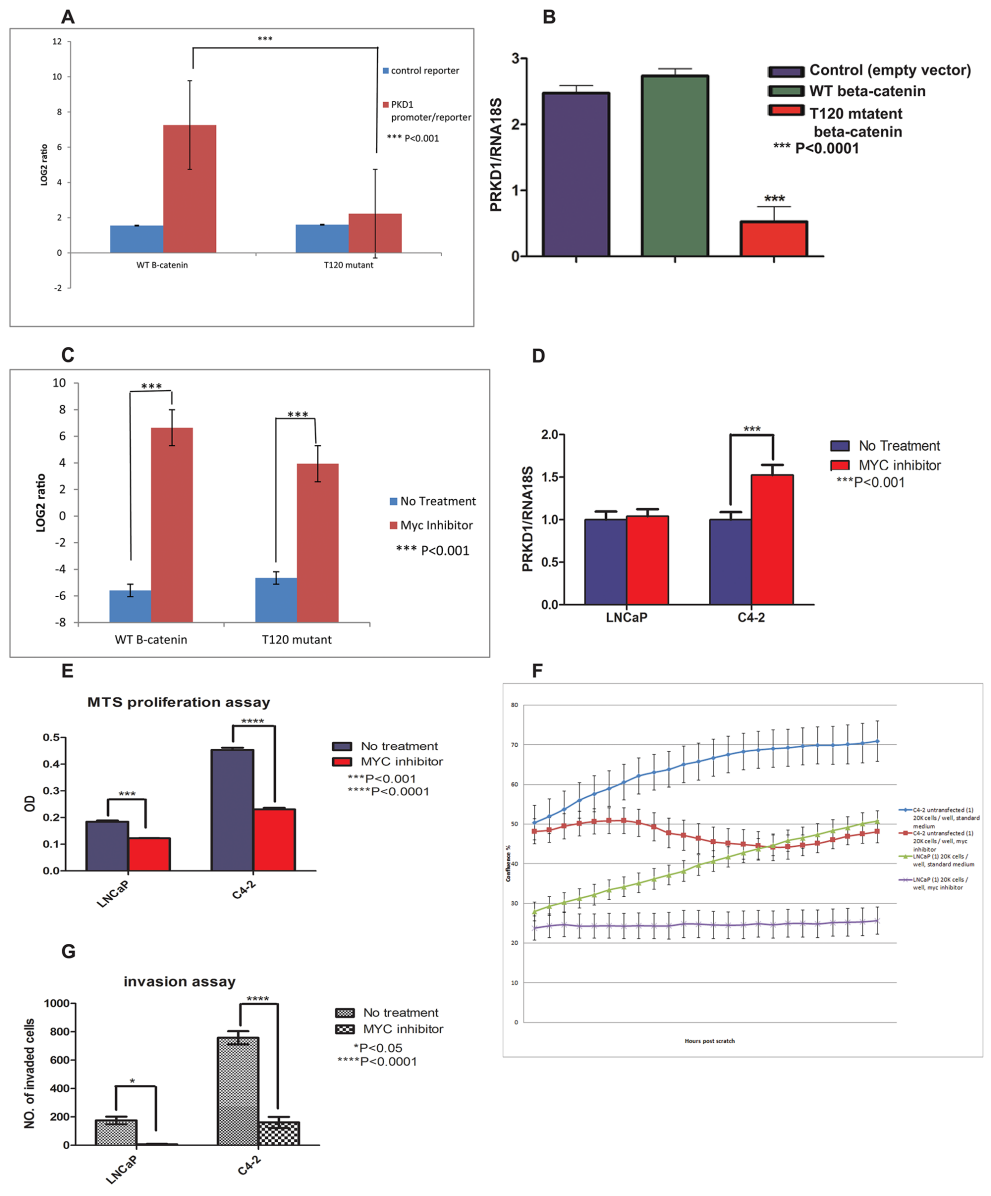
The unphosphorylated threonine 120 (T120) mutant beta-catenin represses *PrKD1* gene expression LNCaP prostate cancer cells transfected with wild type beta-catenin (WT- B-cat), T120 mutated beta-catenin (T120 mu) or empty vector (E). **(A)** The unphosphorylated T120 mutant beta-catenin represses *PrKD1* promoter activity; LNCaP cells co-transfected with beta-catenin constructs (WT beta-catenin, T120-mutant beta-catenin and empty vector) and *PrKD1*/luciferase vector. The non-insert luciferase vector was used as control. Thirty six hours after transfection, 100 μl of the assay reagent was added to each well, the plates were incubated at room temperature for 30 min and read on an illuminometer. The Log2 ratio of the average signal from *PrKD1* promoter/reporter transfected cells divided by average signal from non-targeting control was calculated. The results for WT-beta-catenin transfected cells and T120-mutated cells were normalized against empty vector, which show that the cells transfected with unphosphorylated-T120 mutant beta-catenin have significantly less active *PrKD1* promoter activity. **(B)** *PrKD1* gene expression is significantly lower in LNCaP cells transfected with T120 mutant beta-catenin compared to WT beta-catenin or vector control transfected cells. The transcriptional expression of *PrKD1* was normalized against RNA18S using ddCT. P<0.05 value is considered significant. **(C)** *PrKD1* promoter activity in LNCaP transfected cells in the presence and absence of MYC/MAX dimerization inhibitor demonstrating rescue of *PrKD1* promoter activity by the inhibitor. **(D-G)** The effect of MYC/MAX dimerization inhibitor on high- and low- PrKD1 expressing LNCaP and C4-2 prostate cancer respectively. All experiments were done with or without treatment with 100uM of MYC/MAX dimerization inhibitor. (D) Quantification of PrKD1 transcriptional expression in the presence or absence of MYC inhibitor. Inhibition of MYC resulted in significant increase in the expression of PrKD1 in C4-2 cells. (E) MTS proliferation assay after 72 hours. (F) Migration wound healing assay for 48 hours. 20000 cells were seeded, after an overnight the wound was made using IncuCyte wound maker. Cells incubated in 37°C incubator in the presence or absence of MYC inhibitor. The plate was scanned every 2.5 h hours. Data collection started after an overnight culture. The graph shows the confluence of the cells for 48hours. (G) Matrigel invasion assay; the bar graph shows the number of invaded cells through matrigel coated inserts compared to non-coated control inserts. The invasion ability was significantly lower in C4-2 cells treated with MYC inhibitor.

Having established that loss of PrKD1 expression increases nuclear beta-catenin through loss of T120 phosphorylation (Figure 2A), we next evaluated whether T120 mutant beta-catenin could influence PrKD1 expression. The *PrKD1* promoter activity was measured in LNCaP prostate cancer cells co-transfected with either T120 mutated or wild type beta-catenin (WT) and *PrKD1*-promoter reporter (see Materials and Methods). The results showed a significant decrease in *PrKD1* promoter activity in T120 mutant transfected cells compared to WT beta-catenin transfected cells suggesting that nuclear beta-catenin has a negative regulatory effect on *PrKD*1 promoter (Figure 3A). The results of the reporter assay were corroborated by decreased levels of PrKD1 expression in T120 mutant beta-catenin transfected LNCaP cells compared to wild type control (Figure 3B, Supplementary Figure 3). This negative regulatory effect was rescued by MYC/MAX dimerization inhibitor treatment for 24 hours, which confirmed that the beta-catenin negative regulatory effect is through MYC/MAX complex (Figure 3C). The treatment of C4-2 prostate cancer cells (more invasive derivative of LNCaP cells and with comparatively lower expression of PrKD1) with MYC inhibitor increased the transcriptional expression of PrKD1 confirming the negative regulatory role of MYC on PrKD1 expression (Figure 3D). MYC inhibitor treatment also decreased the proliferation rate, migration and invasive ability of the cells (Figure 3E-3G and Supplementary Videos 1 and 2; the videos show real time wound healing - migration assay- analyzed in Figure 3G).

### PrKD1 down regulation increases AR activity through increasing nuclear beta-catenin

Targeting androgen signaling has been one of the most effective strategies in managing patients with prostate cancer. We had previously shown that whereas increased expression of PrKD1 decreases AR mediated prostate specific antigen (PSA) expression, down regulation of PrKD1 led to AR mediated increased PSA expression [13]. As the current study has demonstrated that PrKD1 increases nuclear beta-catenin and published literature supports beta-catenin as a co-activator of AR, we explored whether the PrKD1 dependent increase in nuclear beta-catenin influenced AR transcriptional activity by studying the expression of a sample of AR responsive genes.

**Figure 4:**
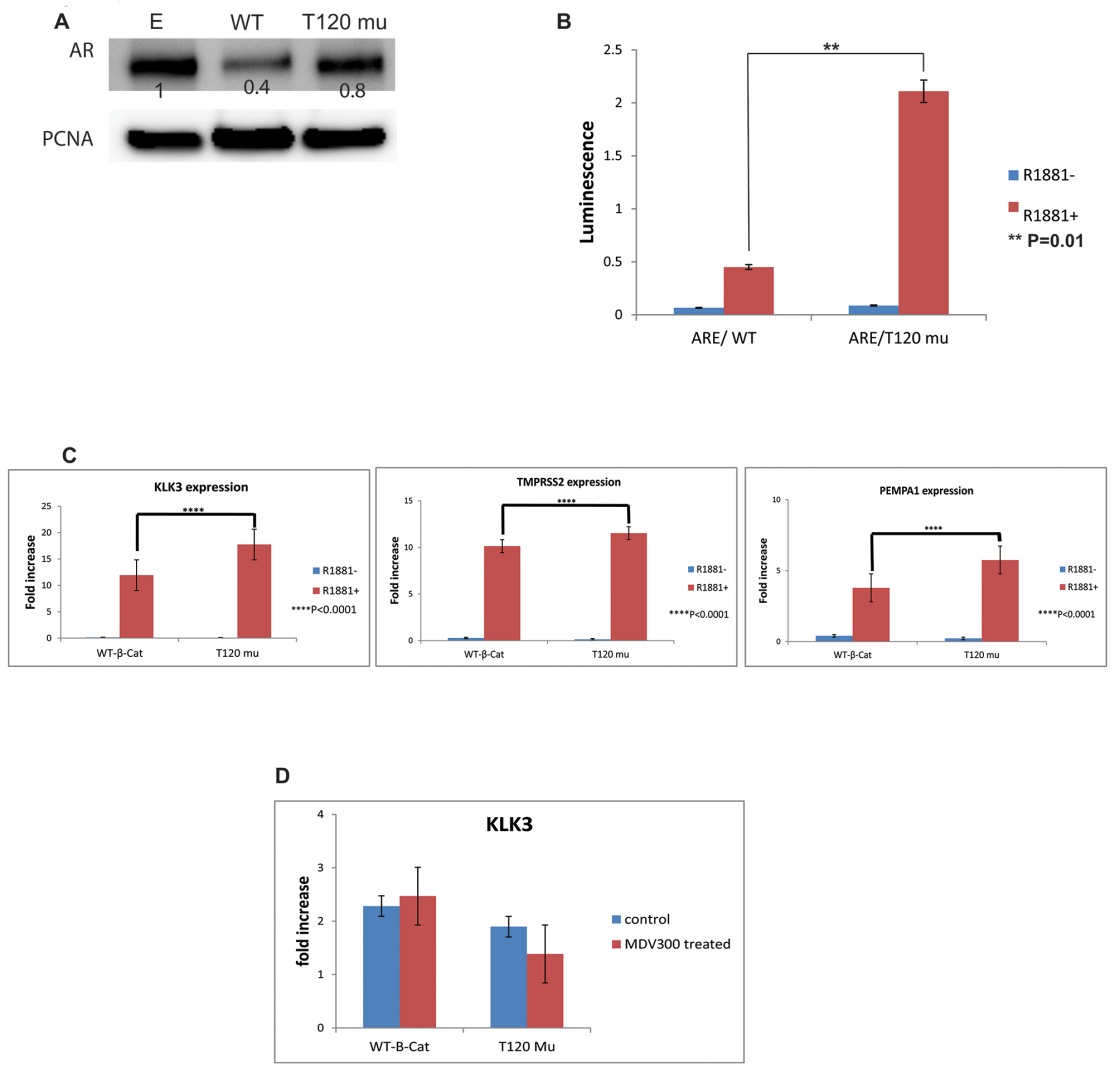
The effect of unphosphorylated T120 mutant beta-catenin on androgen receptor (AR) activity The LNCaP cells were transfected with wild type beta-catenin (WT- B-cat), T120 mutated beta-catenin (T120 mu) or empty vector (E), cultured in charcoal stripped medium with or without 1nM R1881. (**A)** The western blot using nuclear fraction of the transfected cells. The density of each band represented as a relative value normalized against loading control (PCNA). (**B)** AR transcriptional activity using ARE reporter gene in LNCaP transfected cells demonstrating significant activation of AR activity in T120 mutant beta catenin compared to WT-beta-catenin. (**C**) The expression of select androgen responsive genes in response to treatment with R1881. The expression in both WT and mutant beta-catenin transfected samples normalized against the expression in LNCaP cells transfected with empty vector. P values <0.05 is considered as significant. The expression of androgen responsive genes correlates directly with increased AR activity following transfection of T120 beta-catenin mutant compared to WT-beta-catenin control. (**D)** The increased expression of KLK3 (as representative of AR responsive genes) following transfection with T120 mutant beta-catenin was rescued by 10 μM MDV300 (Enzalutamide), suggesting that the T120 beta-catenin mutant effect is mediated by AR. The expression of genes was normalized against the expression of genes in LNCaP cells transfected with empty vector.

In order to study the role of PrKD1 on AR expression and transcriptional activity through changes in beta-catenin phosphorylation and subcellular localization, we used AR reporter assays and cell line transfection experiments. The AR expression did not show significant changes in T120 beta-catenin mutant transfected LNCaP cells compared to control transfected cells (Figure 4A). However, the AR transcriptional activity, and response to androgen were significantly higher in T120 mutant beta-catenin transfected cells compared to WT type control (Figure 4B). Moreover, the expression of three androgen responsive genes, KLK3 (PSA), TMPRSS2 and PEMPA1, was quantified in the presence or absence of androgens, which showed significant increase in expression of AR response genes in the beta-catenin T120 mutant transfected cells compared to controls (Figure 4C) confirming the downstream effects of increased AR activity. The response to androgen could be abrogated by treatment with second-generation AR receptor antagonist Enzalutamide, further confirming the specificity of mediation by AR (Figure 4D). No significant changes were observed in androgen non-responsive genes (data not shown).

### Expression of PrKD1 inversely correlates with MAX expression in human prostate tissue

**Figure 5:**
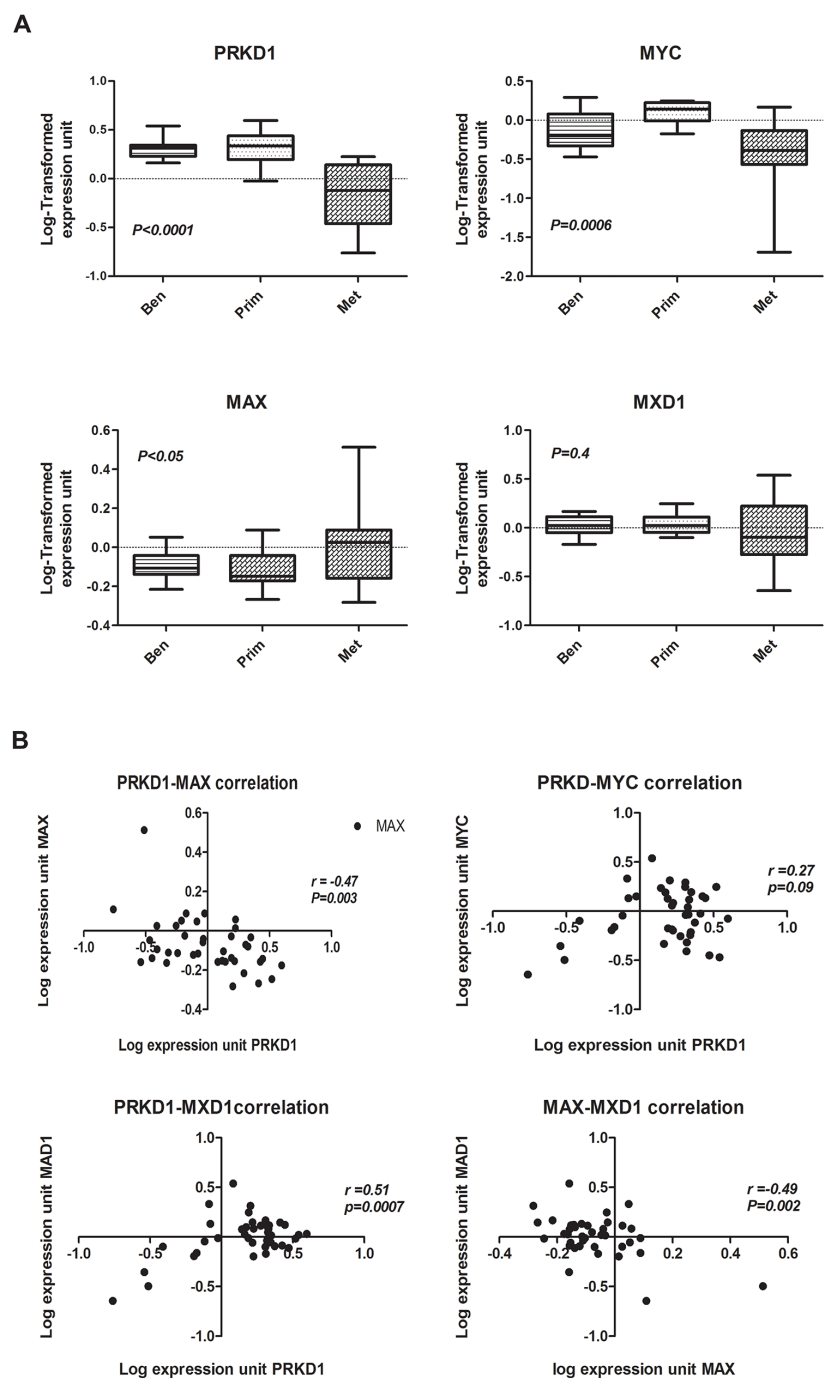
The expression of *MAX* reversely correlates with *PrKD1* in human prostate cancer tissue (**A)** Box plots of *PrKD1*, *MYC*, and *MAX* expression levels in benign (Ben), primary (Prim), and metastatic (Met) human prostate cancer tissue (microarray data set GSE8511). The box represents the interquartile range of data with various samples, and the line through that box represents the median of the distribution. The range is indicated by whiskers on the plot. Benign tumor, *n=16*; primary tumor, *n=12*; metastatic tumor, *n=13*. (**B**) Scattered correlation plots of *PrKD1* versus *MYC*, *MAX*, and *MXD1*; and *MAX* versus *MXD1* for all samples displayed in A.

We interrogated publicly available DNA microarray expression data sets derived from human prostate benign and cancerous tissues using National Center for Biotechnology Information’s Gene Expression Omnibuses [31]. We focused initially on data sets studying benign, primary (localized), and metastatic prostate cancer. Data sets with significant down regulation of PrKD1 in metastatic prostate cancer were identified, and the gene expression data for MYC, MAX and MXD1 were compared (Figure 5). At a molecular level, whereas **MYC**/MAX heterodimerization leads to activation of MYC mediated transcription, heterodimerization of **MXD1**/MAX leads to inhibition of MYC activity [14]. Our analysis showed that the expression of MAX (and not MYC) inversely correlated (r=-0.47, P=0.003) with PrKD1. As expected, the expression of MXD1 directly correlated with PrKD1 (r=0.51, p=0.0007) and inversely with MAX expression (r=-0.49, p=0.002), which suggests that the heterodimerization of MAX with either MYC or MXD1 can down or up regulate *PrKD1* gene expression through activation or inhibition of MYC activity respectively. Beta-catenin was not included because we have shown that only subcellular nuclear translocation of beta-catenin, and not changes in the beta-catenin gene expression, are involved in *PrKD1* regulation.

## DISCUSSION

While PrKD1 is up regulated in pancreatic and skin cancers, it is down regulated in breast, gastrointestinal and advanced prostate cancers [2]. Whereas the pro- and anti-oncogenic roles of Protein Kinase D1 (PrKD1) depending on the type of cancer is well recognized, the regulatory mechanisms of *PrKD1* gene expression seems varied among the cancer types. There is paucity of data regarding regulation of *PrKD1* gene expression in the published literature other than DNA methylation being involved in *PrKD1* down regulation in gastric cancers and by nuclear beta-catenin in colon cancer [2, 15]. In this study we have identified MYC/MAX and beta-catenin as a novel transcriptional complex involved in regulation of PrKD1 gene expression.

MYC is a well-studied transcription factor known to be involved in cell proliferation and apoptosis. Whereas the transcriptional activity of MYC is induced by obligate heterodimerization with MYC-associated protein X (MAX), the heterodimerization of MAX with MAX dimerization protein 1 axis (MXD1 or MAD1) inhibits MYC transcriptional activity [14]. The MYC transcription factor binds to E-box sequence 5’-CAC(G/A)TG-3’ and becomes transcriptionally active only after obligate dimerization with MAX [9, 16]. While there are over 40 known MYC target genes in the published literature [17], we have identified for the first time that *PrKD1* is a target gene under MYC transcriptional control. In fact, the *PrKD1* gene contains the consensual sequence for MYC/MAX binding in several regions including the promoter region and further investigation in the area can elucidate the effects of MYC binding to other *PrKD1* binding sites. In addition, the function of MYC is targeted by several upstream proteins including receptor tyrosine kinase stimulated Ras/Raf signaling and PI3K (phosphoinositide 3-kinase) mediated AKT signaling pathways. Interestingly, the *MYC* gene has several Wnt responsive elements and one of the upstream regulators of MYC is nuclear beta-catenin, which is increased by down regulation of PrKD1. The complex network of mutually dependent protein expression and activity suggests built in auto-regulatory mechanisms to regulate critical cellular functions, and nuclear beta-catenin is a key player in the study network.

Beta-catenin is generally thought of as a transcriptional coactivator that is recruited to DNA sites called Wnt responsive elements by T-cell factor/Lymphoid enhancer factor (TCF/LEF) family of sequence-specific transcription factors [18]. Although the *PrKD1* gene has several Wnt responsive DNA elements, the beta-catenin did not bind to the putative TCF/LEF factor in the transcription array (cell E6 in Supplementary Figure 1 and Figure 1C) suggesting beta-catenin represses *PrKD1* expression by non-canonical pathway. Because MYC/MAX is the only transcription factor from the array that binds to the same site as beta-catenin on the *PrKD1* promoter, the MYC/MAX is identified as a novel transcription factor mediating the non-canonical gene repression by beta-catenin [19]. While there is no published data on binding of beta-catenin to MYC/MAX transcription factors, predictive modeling suggests a plausible interaction [20, 21]. On the other hand, beta-catenin and MYC/MAX could interact via intermediary factors. While we have convincingly shown that the negative regulatory effect of beta-catenin/MYC/MAX complex on *PrKD1* promoter, the details of other co-factors and sequence of interaction leading to nuclear beta-catenin/MYC/MAX transcription complex influence on *PrKD1* expression remains to be understood. More recently, there has been improved understanding of co-repressor functions of beta-catenin as well [22] and that PrKD1 is yet another beta-catenin target for repression. We have previously shown that down regulation of PrKD1 is associated with prostate cancer progression through several molecular mechanisms [2, 8, 13, 23, 24] including nuclear translocation of beta-catenin and by influencing AR transcriptional activity.

AR is a key driver of prostate cancer progression and has been an effective therapeutic target in managing patients with advanced prostate cancer [25]. The active nuclear beta-catenin increases AR transcriptional activity without altering the expression of AR [26]. The down regulation of PrKD1 in advanced prostate cancer increases AR activity through at least two distinct mechanisms. Firstly, PrKD1 is known to phosphorylate heat shock protein 27 (Hsp27) at Serine 82, a AR nuclear transporter [27], represses AR transcriptional activity [24] and loss of PrKD1 increases AR nuclear translocation by decreasing serine-82 phosphorylation of Hsp27. Secondly, loss of PrKD1 results in reduced phosphorylation of T120 in beta-catenin resulting in increased nuclear beta-catenin and the AR co-activation [7, 27, 28]. These findings are consistent with published literature that Wnt/beta-catenin pathway is one of the compensatory pathways activated in prostate cancer in response to androgen deprivation therapy [29]. In this study, the novel observation is recruitment of beta-catenin to *PrKD1* promoter site. Interestingly, the recruitment of beta-catenin is independent of *TCF4* and *AR*, which have overlapping interaction domain on beta-catenin and demonstrate competitive binding [7].

**Figure 6:**
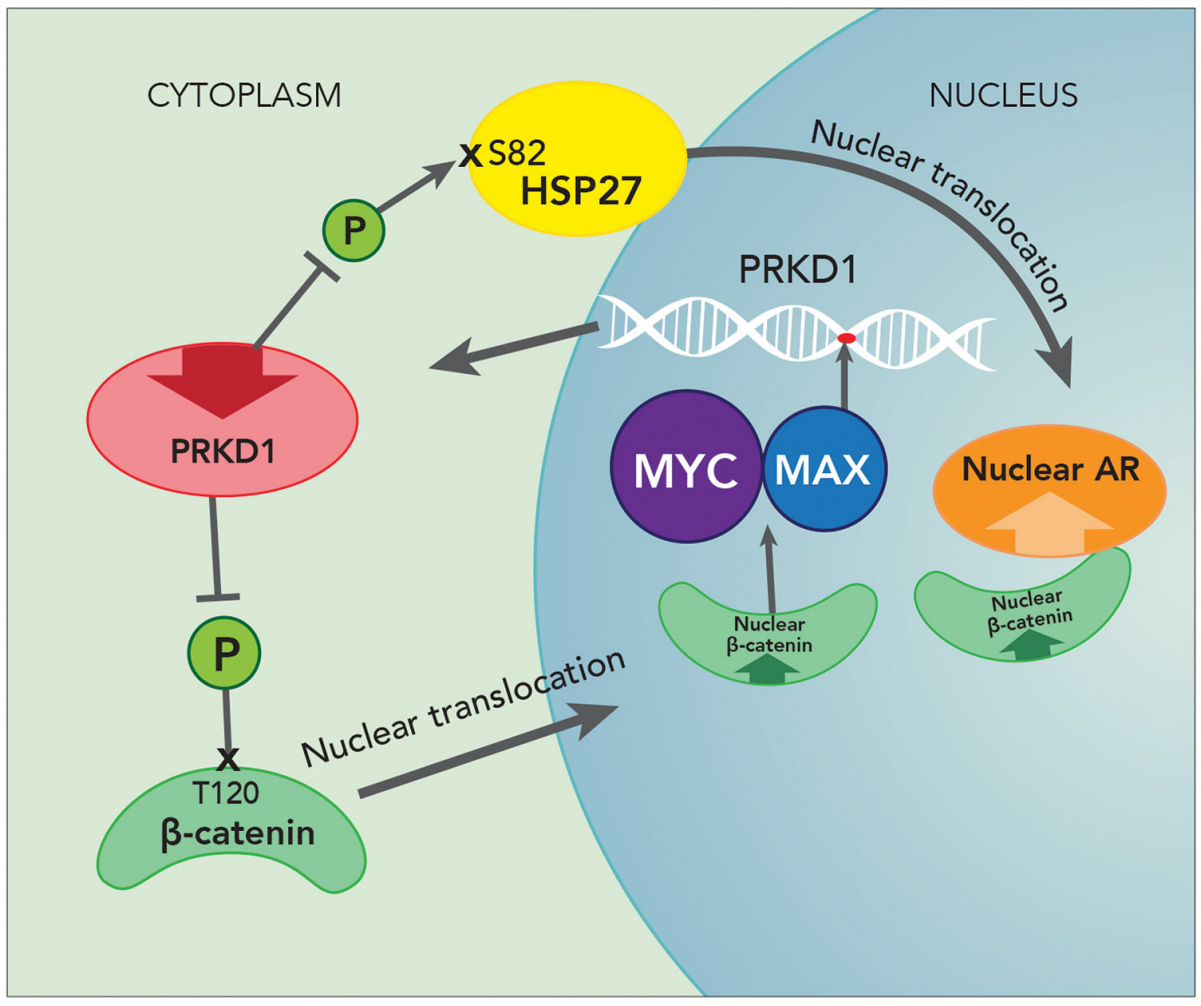
Auto repressive loop of PrKD1 down regulation in prostate cancer PrKD1 is down regulated in advanced prostate cancer, which decreases phosphorylation of beta-catenin at thereonine120 (T120) residue. The T120 unphosphorylated beta-catenin translocates to the nucleus resulting in increased nuclear beta-catenin activity. The nuclear beta-catenin in turn represses *PrKD1* expression through MYC/MAX complex and thereby perpetuating down regulation of PrKD1, which is associated with prostate cancer progression. Increased nuclear beta-catenin resulted in increased AR coactivation. Down regulation of PrKD1 decreases phosphorylation of HSP27 at Serine 82 (S82) which facilitates and increases AR nuclear translocation (see discussion).

Based on the complex interplay between PrKD1, AR, beta-catenin and MYC/MAX, we rationalize an auto-repressive loop that regulates PrKD1 expression. While the precise initiating mechanism leading to down regulation of PrKD1 in prostate cancer is unclear, the down regulated PrKD1 leads to loss of T120 phosphorylation of beta-catenin, which increases active nuclear beta-catenin with attendant consequences on downstream transcriptional activity. The increased active nuclear beta-catenin through interaction with MYC/MAX transcription factor functions as a co-repressor of *PrKD1* expression and thereby perpetuates the down regulation of PrKD1 in advanced prostate cancer (Figure 6). The down regulation of PrKD1, among other mechanisms, contributes to increased AR activity and progression of prostate cancer including castration resistance.

The down regulation of PrKD1 and improved understanding of its role in prostate cancer progression makes PrKD1 an attractive target for therapeutics and biomarker development. We have published a PrKD1 centered signaling biomarker panel that can be used to discriminate aggressive from indolent prostate cancer [11]. The addition of MYC/MAX to the panel may improve the sensitivity and specificity, and thus improve chances of utilization in clinical settings. A better understanding of molecular regulation of PrKD1 allows for more effective therapeutic targeting. While additional studies are needed to prove the feasibility and efficacy of targeting in prostate cancer, it is conceivable that targeting the transcriptional regulators such as MYC/MAX could selectively and indirectly increase the PrKD1 expression in cells.

In summary, the current study for the first time has investigated transcriptional regulation of *PrKD1* and identified *PrKD1* as a transcriptional target for MYC/MAX and beta-catenin transcription complex. The interaction creates a novel auto-repressive loop that perpetuates PrKD1 down regulation in prostate cancer. Because PrKD1 down regulation in advanced prostate cancer contributes to prostate cancer progression and castration resistance by increasing AR transcriptional activity, therapeutic targeting of PrKD1 through transcriptional regulation is a novel possibility. Moreover, addition of MYC/MAX to PrKD1 centered biomarker panel may improve the performance of the panel in discriminating indolent from aggressive prostate cancer. Future detailed mechanistic studies will clarify balancing role of MYC/MAX and MYC/MAX/MXD1 axis in *PrKD1* expression.

## MATERIALS AND METHODS

### Cell culture and transfection

Prostate cancer cell lines, LNCaP (ATCC) and its more metastatic and less androgen dependent derivative, C4-2 cells (Urocor), were cultured in RPMI 1640 medium plus 10% Fetal Bovine serum (FBS, Invitrogen). For androgen treatment and control, charcoal stripped serum (CSS, Gemini, #100119) was used instead of FBS, with or without R1881 (Sigma-Aldrich, R0908), using 1 nM R1881 for androgen treatment. Enzalutamide (MDV300, MedChem, # HY-70002), a second generation AR antagonist, at 10 μM final concentration, was used for AR inhibition. LNCaP cells were stably co-transfected with either wild type beta-catenin (WT), T120 mutated beta-catenin (T120 mutant) or empty vector (DsRed2-C1) constructs as previously described [10]. The transfection was performed using incubation of cells with Fugene HD transfection reagent (Promega, #E2311) overnight, recovered for 24 hours in complete growth medium and selected with 400 μg/ml G418 (Clontech, # 631307) for a week. All experiments described were done per manufacturer’s protocols when commercially available kits were used. The stably transfected C4-2 cells with *PrKD1-GFP* (C4-2/*PrKD1*) used in the Migration experiment (Figure 3F) were previously described [30]. For MYC inhibition treatment, the cells were treated with 10058-F4, a MYC inhibitor that inhibits the c-MYC/MAX interaction, (Sigma, # F3680), for at least 24 hours at 100 μM concentration.

### Measuring AR transcriptional activity

The non-transfected and stably transfected cells were seeded in 96 well plates. The cells were co-transfected with ARE-dual luciferase formatted reporters gene using Cignal reporter assay kit (Quiagen, CCS1019L). The co-transfected cells were recovered in growth medium (RPMI1640+ CSS 10%) with or without androgen for 24 hours. The luciferase assay was developed using Dual-Luciferase Reporter Assay system (Promega, E2920). The AR activity was calculated as the ratio of Luciferase/Renilla Bioluminescence. For beta-catenin transfected cells, the results were normalized against empty vector-transfected cells. All transfections were performed in triplicates. Positive and negative controls (provided in the kit) were included in each experiment.

### Measuring protein kinase D1 gene (PrKD1) promoter activity

LightSwitch/*PrKD1* promoter reporter construct (Active Motif, # S710918) was co- transfected with beta-catenin constructs (WT and T120 mutant) in LNCaP cells using Fugene reagent. After an overnight co-transfection and 24 hour recovery, a luciferase assay was performed on co-transfected LNCaP cells using LightSwitch Luciferase Assay kit (Switchgear Genomics, #LS010). The measured luminescence was representative of *PrKD1* promoter activity in each cell line. Luminescence in transfected cells were normalized against the empty vector.

### Quantitative real time PCR (qPCR)

The RNA was extracted from the cell lines using RNeasy Plus Mini Kit (Qiagen, 74134). Four μg of RNA was used for cDNA synthesis using Transcriptase III (Invitrogen, 18080). Primer sets for androgen response genes and controls were designed using NCBI primer pick tool for Sybr green assays (Supplementary Table 1) or selected and ordered from ABI TaqMan assays (Applied Biosciences; Supplementary Table 1). For RNA expression quantification, primers with the best coverage and probes that spanned exons were selected. qPCR was performed using 50 ng cDNA from cultured prostate cancer cells in the presence of one μl of primer set (10 μM) and 10 μl of Taqman Universal PCR MasterMix (Applied Biosystem, 4304437) or Sybr Select MasterMix (Applied Biosystems, Life technologies, 4472908) in 7300 real time PCR system (Applied Biosystems). RNA 18S was used as housekeeping gene for normalization using the ddCT method.

### Chromatin immunoprecipitation (CHIP) assay

The CHIP assay was performed using Simple CHIP kit (Cell Signaling, #9003). Briefly, cultured cells were incubated with 37% fomaldehyde to crosslink proteins to DNA for 10 minutes, collected and centrifuged after adding glycine. After nuclear extract preparation, the chromatin was digested using Micrococcal Nuclease, immunoprecipitated overnight with beta-catenin (Santa Cruz, #SC7199), MYC (cell signaling, #9402) or MAX (Cell Signaling, #4739) antibodies followed by elution through CHIP-Grade protein G Magnetic Beads (Signosis, # 2729) for 2 hours. Antibody against histone H3 (Cell Signaling, #4620) was used as positive control, mouse and rabbit IgGs as negative controls. Chromatin was eluted from the antibody/protein G complex using SDS containing elution buffer. DNA was isolated and quantified by qPCR using promoter-specific primers (Supplementary Table 1).

### Chromatin immunoprecipitation (CHIP) sequencing

Part of the DNA prepared (pulled down with either beta-catenin or IgG) for CHIP assay was used in CHIP sequencing. DNA sequencing was performed on an Illumina NextSeq 500 sequencer in the Wake Forest Cancer Genomic Core Laboratory. Each DNA library was barcoded to allow optimized pooling in order to reduce sequencing cost. We carried out single-end DNA sequencing at up to 75 base pair (bp) single end, which was determined based on estimated fragment capture sizes. At the 75 bp scale and indexing of 12 libraries per flow cell, we had the capacity to generate up to 30 million sequencing tags/sample with an output of >∼ 26 gigabase per flow cell.

### Data mining, bioinformatic and statistical analysis

The data set (GSE8511) with significant down regulation of PrKD1 in metastatic samples, was downloaded and analyzed using the statistical language R [31, 32]. All data were log transformed using the robust multichip average algorithm. The normalized expression values were used to plot the data. The scatter plot was overlaid on linear regression lines. For CHIP sequencing analysis, all DNA sequence was aligned to the human reference genome (GRCh38/38) using standard BWA based aligners. Downstream analysis peak calling was performed using the software pack MACS (model-based analysis of ChIP-Seq) [33]. MACS reports genomic regions enriched for Top1 binding by comparing the experimental sample to a control sample and empirically determines false discovery rate. We used ‘input’ DNA as control since an ‘IgG control’ (anti-Top1 mAb (Abcam; Ab3378)) library could be biased due to lack of sufficient sequence complexity [34]. During data analysis, we required a MACS P-value <1e-5 for calling the Top1 peaks.

### PrKD1-Knock out (KO) mouse strain

Generation of prostate specific *PrKD1* Knock down (with genotype of PB-Cre4; *PrKD1*^lox/lox^) mice was described previously [12]. *PrKD1* knock-out efficiency was confirmed by mRNA expression and at protein level [12]. Male mice with PB-Cre4/PrKD1^lox/Lox^ genotype were used as *PrKD1* KO and animals without PB-Cre4 used as control because they lack PrKD1 down regulation [12]. Mouse euthanasia was done by CO_2_ inhalation followed by cervical dislocation (secondary method). The studies were done following approval by the Institutional Animal Care and Use Committees (IACUC).

### Promoter binding transcription factor (TF) profiling

The potential activity of 48 transcription factors (TFs) were measured using TF activation plate array 1 (Signosis, #FA1001) for promoter binding TF profiling. Briefly, nuclear extract of LNCaP cells (pulled down with beta-catenin) with or without *PrKD1*-promoter oligonucleotide were incubated with a biotin tagged probe mix to generate TF/probe complex. After spin column purification to separate un-bounded probes from complexes, the captured probes are then detected with Streptavidin-HRP and chemiluminescent substrate. *PrKD1* promoter region regulated by beta-catenin was amplified by real-time PCR and the amplified region was used as *PrKD1*-promoter-specific-oligonucleutide. If the DNA fragment contains a TF binding sequence, it will compete for the biotin-labeled probe to bind to the TF in the sample. When the changes in reading between two samples were ≥1.5 fold, the results were considered significant for activated TFs.

### Immunohistochemical and fluorescent staining

Immunohistochemical detection of beta-catenin, and PrKD1 was performed on mouse prostate tissue and transfected and non-transfected cell lines using anti-beta-catenin (Santa Cruz, #SC1496) and anti PKC mu (Santa Cruz, # 693)^8^. Mouse prostate tissue was fixed in 4% paraformaldehyde. The paraffin-embedded paraformaldehyde-fixed mice tissue was cut at 5 micron thickness. The first slice of staining sections was used for histological confirmation by Hematoxylin and Eosin (H&E) staining. The tissue slides and fixed cells were treated with 0.1% Triton-X-100 for 3 min, followed by blocking nonspecific binding using protein block solution (Dako, #X0909), overnight incubation with primary antibody at 4°C and one hour incubation with fluorescent secondary antibodies AF594 (Life technologies #SA5-10088) or AF488 (life technologies, #A11070) and contra-stained with DAPI for 5 minutes. Goat isotype IgG (Santa Cruz) was used as negative controls. Tissue slides were examined using Olympus Fluoview (FV10i) confocal microscope and cells were analyzed by IN Cell analyzer 2000 cell imaging system (GE Healthcare Life Sciences).

### Western blot

The nuclear proteins were extracted from different cell lines using nuclear extraction kit (Signosis, #SK-0001); whole cell lysate was extracted using Pierce RIPA buffer (Thermo Scientific, 89900) and other subcellular proteins were extracted using subcellular protein fractionation kit for cultured cells (Thermo Scientific, QE216067C). Lysates containing equal amounts of protein were separated on 10% SDS-PAGE gels and transferred onto a PVDF membrane (Millipore, Billerica, MA). All primary antibodies were obtained from Santa Cruz Biotechnologies. Total protein loading was determined by probing the membranes for beta-actin or PCNA. The bands were visualized using horse-radish peroxidase (HRP) conjugated secondary anti-mouse or anti-rabbit antibody (Cell Signaling Technology) in conjunction with chemiluminescence substrate (34094, Thermo Scientific) via LAS 3000 imaging system (Fuji Photo Film). Image J software was used to analyze the density of electrophoretic western blot bands. The values were normalized against beta-actin or PCNA expression.

### MTS proliferation assay

Approximately 3 ×10^3^ cells per well were seeded into 96-well plates. After overnight incubation, medium was changed to treatment (MYC inhibitor 100 μM) in half of the wells. Cell proliferation was evaluated after 72 hours by incubating cells in MTS/PMS mix (CellTiter 96 Aqueous MTS reagent, G1111, Promega/phenazine methosulfate, P9625, Sigma) for 1 hour at 37° C. Absorbance was detected at 490 nm with a microplate reader (SpectraMax M5, Molecular Devices). The culture medium was used as a blank. Experiments were done in triplicate. Cell viability in different treatment groups compared using two-way analysis of variance (ANOVA) with Bonferroni post-test correction.

### Invasion assay

Cells were seeded in Matrigel coated cell culture inserts (BD, 24 well, # 353097) and non-coated inserts (as control) in serum free growth medium with or without MYC inhibitor treatment at 2.5 x 10^4^ cells per insert. Growth medium with 10% FBS was used as chemoattractant. After 24 hours, the cells on the lower surface of the membrane were stained with Diff-Quick stain following the manufacturer’s protocol. The invading cells were counted using 10X magnification on a Zeiss imager M1 microscope. The percentage of invading cells for each of the conditions was calculated as the mean number of invading cells through Matrigel coated inserts divided by mean number of cells migrated through uncoated insert membrane. The experiments were done in triplicates.

### Migration assay

The cells were seeded on a black/clear Falcon 96 well plate at 2 x 10^4^ per well. A scratch wound was produced using IncuCyte wound maker (Essen bioscience, #4493). After two washes, cells were cultured in standard growth medium in the presence or absence of MYC inhibitor treatment for 96 hours and migration is captured for 48 hours by two hour scans intervals in IncuCyte ZOOM system and analyzed briefly using ZOOM software.

## SUPPLEMENTARY MATERIALS FIGURES, VIDEOS AND TABLES







## Abbreviations

**PrKD1**: protein kinase D1
**T120**: threonine at position 120
**WT**: wild type
E: empty vector
**TF**: transcription factor
**KO**: knock out
**CHIP**: chromatin immunoprecipitation
**bp**: base pair
