# Supplementary material for: Beta-catenin represses protein kinase D1 gene expression by non-canonical pathway through MYC/MAX transcription complex in prostate cancer
Source: Oncotarget. 2017 Aug 12;8(45):78811–24. doi: 10.18632/oncotarget.20229 (PMC5668000; doi:10.18632/oncotarget.20229)
Supplement: Supplementary file 1 [file oncotarget-08-78811-s001.pdf]

# Beta-catenin represses protein kinase D1 gene expression by non-canonical pathway through MYC/MAX transcription complex in prostate cancer

## SUPPLEMENTARY MATERIALS

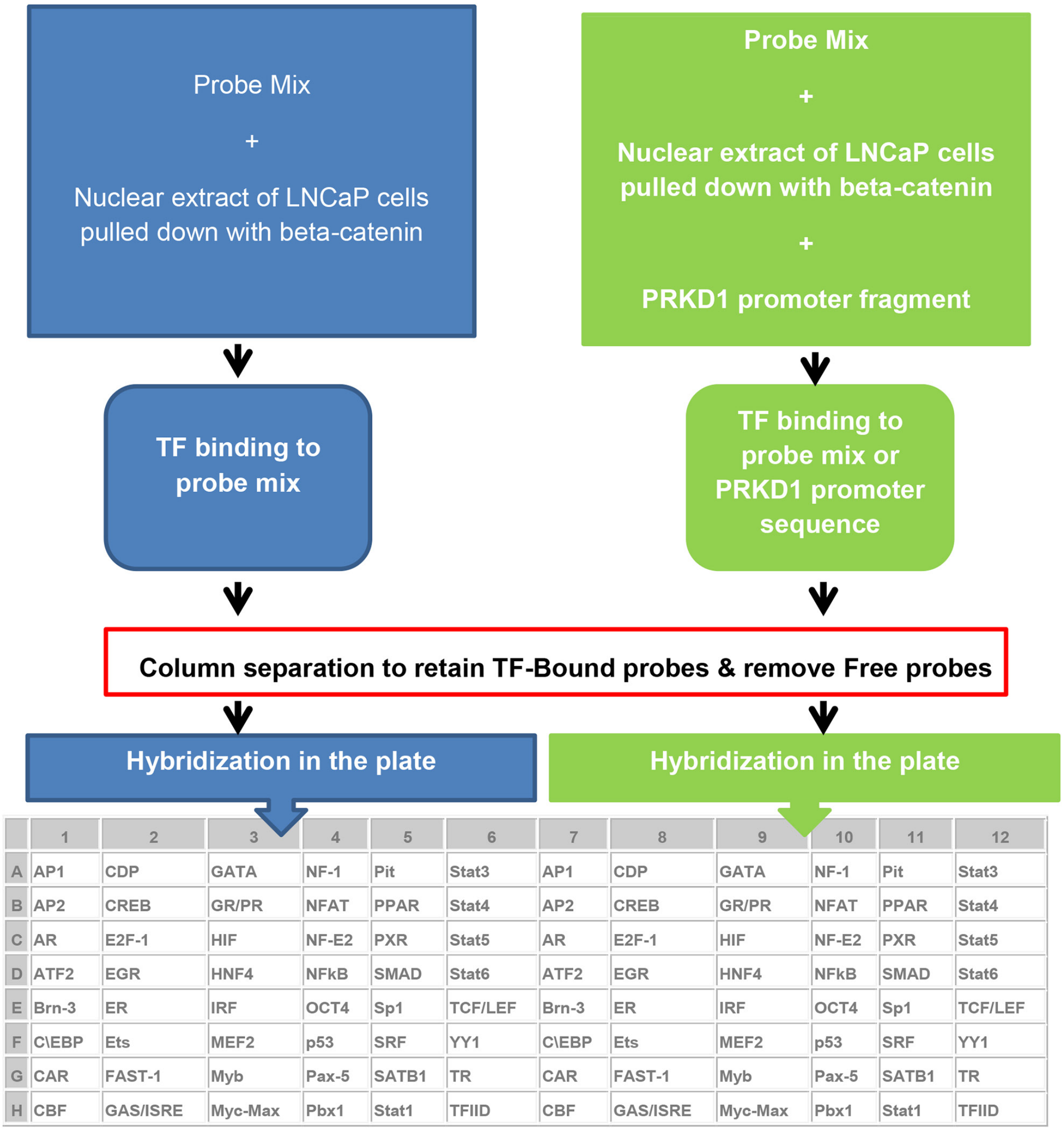


| | 1 | 2 | 3 | 4 | 5 | 6 | 7 | 8 | 9 | 10 | 11 | 12 |
|---|---|---|---|---|---|---|---|---|---|---|---|---|
| A | AP1 | CDP | GATA | NF-1 | Pit | Stat3 | AP1 | CDP | GATA | NF-1 | Pit | Stat3 |
| B | AP2 | CREB | GR/PR | NFAT | PPAR | Stat4 | AP2 | CREB | GR/PR | NFAT | PPAR | Stat4 |
| C | AR | E2F-1 | HIF | NF-E2 | PXR | Stat5 | AR | E2F-1 | HIF | NF-E2 | PXR | Stat5 |
| D | ATF2 | EGR | HNF4 | NFkB | SMAD | Stat6 | ATF2 | EGR | HNF4 | NFkB | SMAD | Stat6 |
| E | Brn-3 | ER | IRF | OCT4 | Sp1 | TCF/LEF | Brn-3 | ER | IRF | OCT4 | Sp1 | TCF/LEF |
| F | C\EBP | Ets | MEF2 | p53 | SRF | YY1 | C\EBP | Ets | MEF2 | p53 | SRF | YY1 |
| G | CAR | FAST-1 | Myb | Pax-5 | SATB1 | TR | CAR | FAST-1 | Myb | Pax-5 | SATB1 | TR |
| H | CBF | GAS/ISRE | Myc-Max | Pbx1 | Stat1 | TFIID | CBF | GAS/ISRE | Myc-Max | Pbx1 | Stat1 | TFIID |

**Supplementary Figure 1: Transcrioption Factor (TF) array design.** Probe mix contains biotin labeled oligos corresponding TFs that are defined for the array. TFs exist in the nuclear extract attach to corresponding probes. Alternatively, if PRKD1 promoter sequence is present in the mix, the TFs that regulates PrKD1 will attach to PrKD1 sequence and not to the corresponding probes. After a simple spin separation of the complexes from unbound free biotin-labeled oligos with a membrane-based column, TF-bound oligos eluted from the column and used for plate hybridization in which complementary DNA of biotin-labeled oligos were pre-loaded. The captured oligo is then detected with streptavidin-HRP and a chemiluminescent substrate. If unlabeled PrKD1 promoter DNA fragment contains a TF binding sequence, it competes with the biotin-labeled oligo to bind to the TF in the sample, leading to no or less biotin labeled TF/DNA complex formation and no or lower detection. Through comparison in the presence and absence of the competitor promoter DNA fragment, promoter-bound TFs can be identified. This design is based on http://www.signosisinc.com/principle/Promoter_Binding_TF_Profiling_Plate_Array.

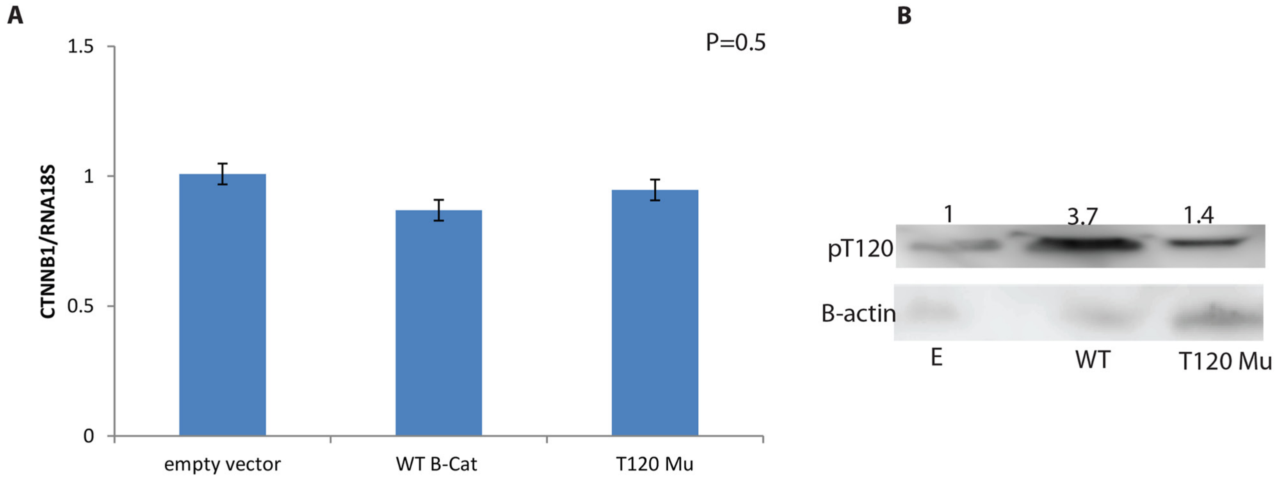


**Supplementary Figure 2: LNCaP cells transfected with wild type beta-catenin and T120 mutant beta-catenin show the expected characteristics.** **(A)** The RNA expression of beta-catenin in LNCaP transfected cells did not show any significant changes which rules out the dominant negative effect of the transfection Process. Column 1(Empty vector): LNCaP cells transfected with empty backbone of the vector; column 2 (WT B-Cat): LNCaP cells transfected with wild type beta-catenin; column 3 (T120 Mu): LNCaP cells transfected with T120 mutant of beta-catenin. (**B)** Western blot analysis in LNCaP transfected cells. Lane 1 (E): LNCaP cells transfected with empty backbone of the vector; Lane 2 (WT): LNCaP cells transfected with wild type beta-catenin; Lane 3 (T120 Mu): LNCaP cells transfected with T120 mutant of beta-catenin. Density of each band normalized against beta-actin (loading control) and corrected for transfection effect against LNCaP cells transfected with empty back bone. pT120: Antibody for phosphorylated beta-catenin at T120 residue.

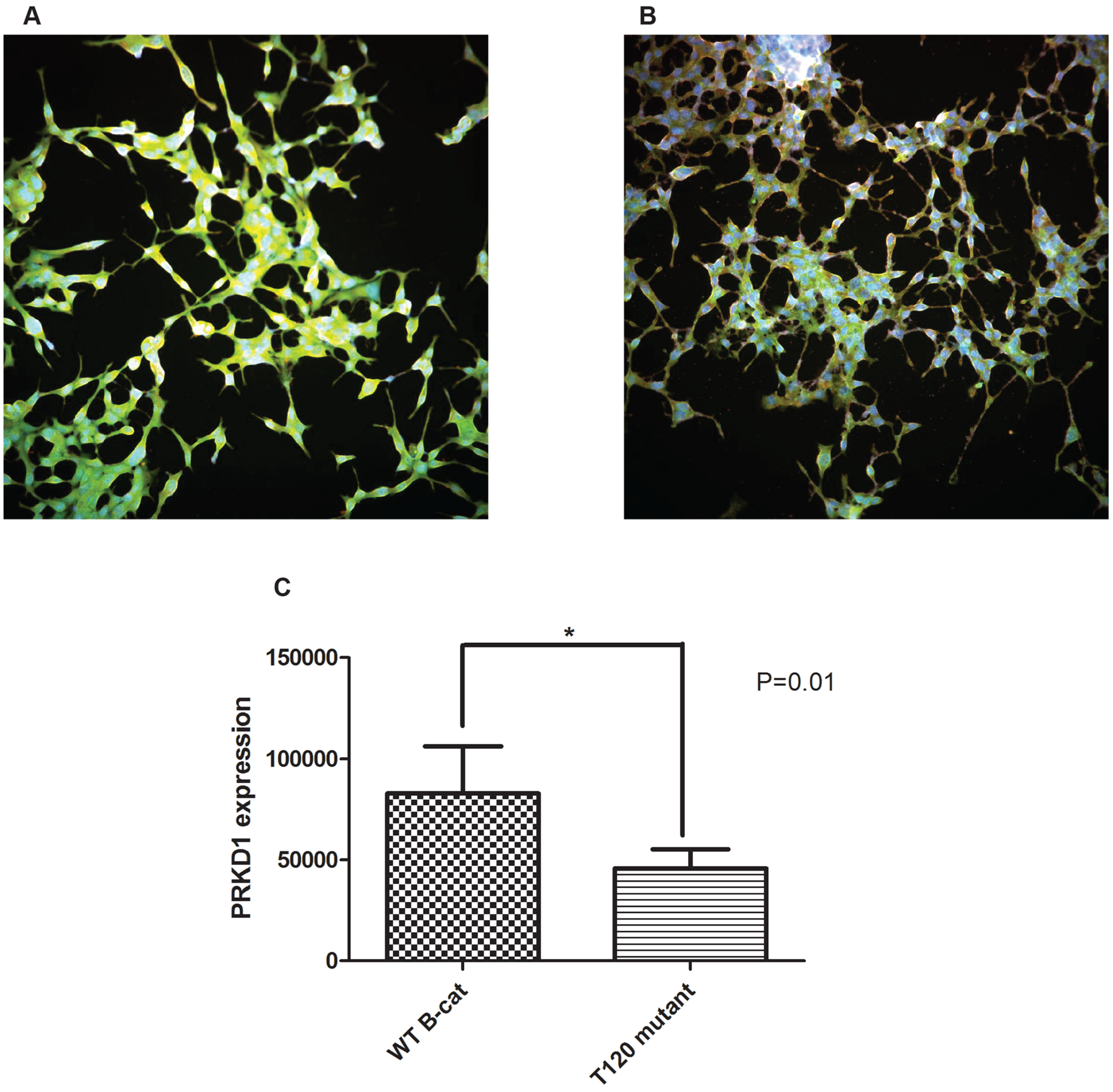


**Supplementary Figure 3: T120 mutation of beta-catenin decreases PrKD1 expression.** LNCaP cells transfected with WT beta-catenin, T120 mutant or empty vector, Fixed and stained for active beta-catenin (red) and PrKD1 (green). Dapi (blue) used for nuclear contra-staining. Stained cells were analyzed in InCell analyzer imaging system. **(A)** Representative of staining of LNCaP cells transfected with WT beta-catenin which shows colocalization of active beta-catenin (in cytoplasm and membrane) and PrKD1. **(B)** Representative of staining of LNCaP cells transfected with T120 mutant which show decreased PrKD1 expression but not active beta-catenin. **(C)** Graph bar compares the intensity of Green fluorescent (representing PrKD1) in samples. The intensity in both samples is corrected against empty vector.

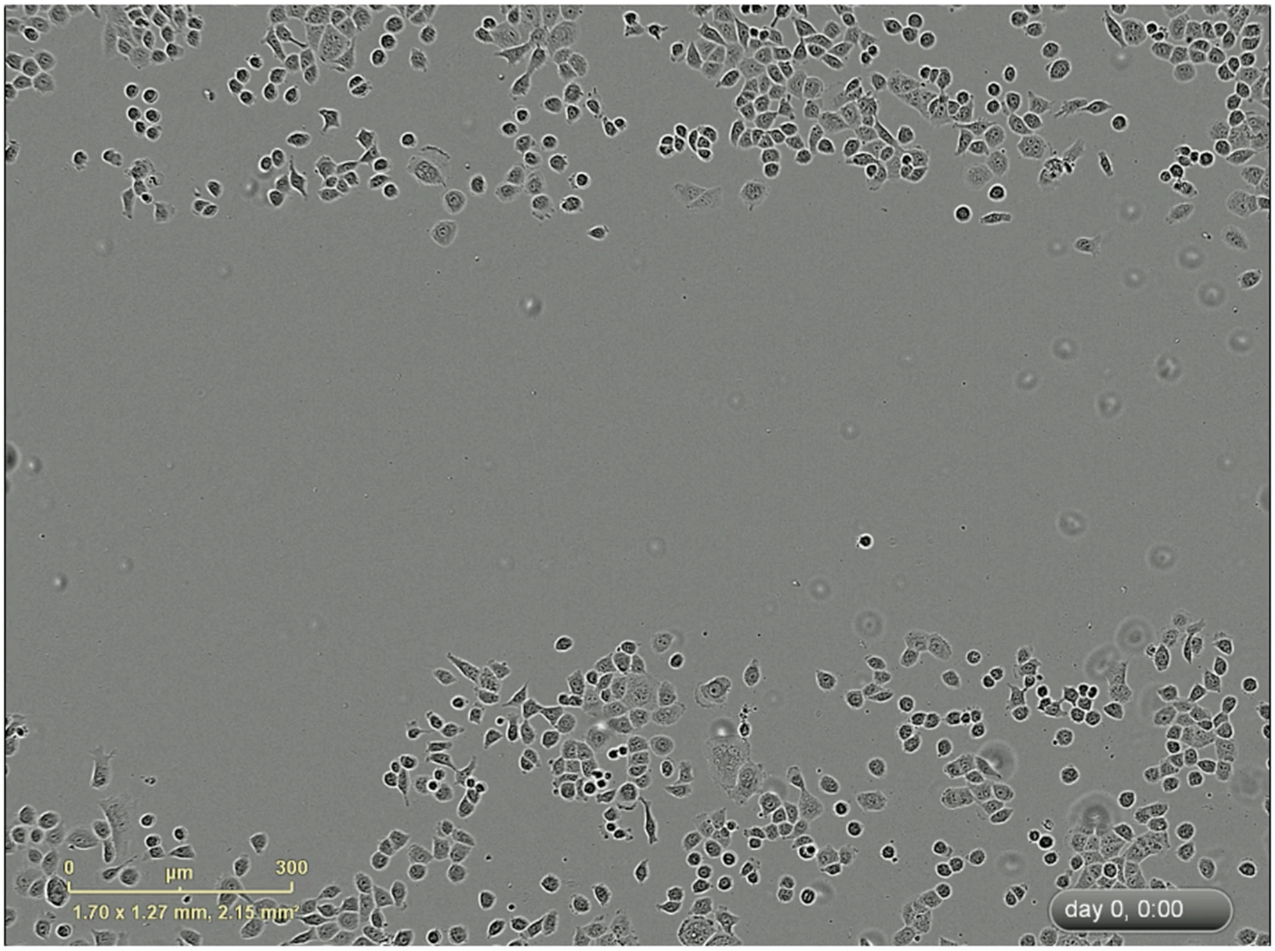


**Supplementary Video 1: Related to Figure 3G - Wound healing assay for C4-2 prostate cancer cells(with low expression of PrKD1)in the presence of MYC inhibitor.**

**See Supplementary Video 1**

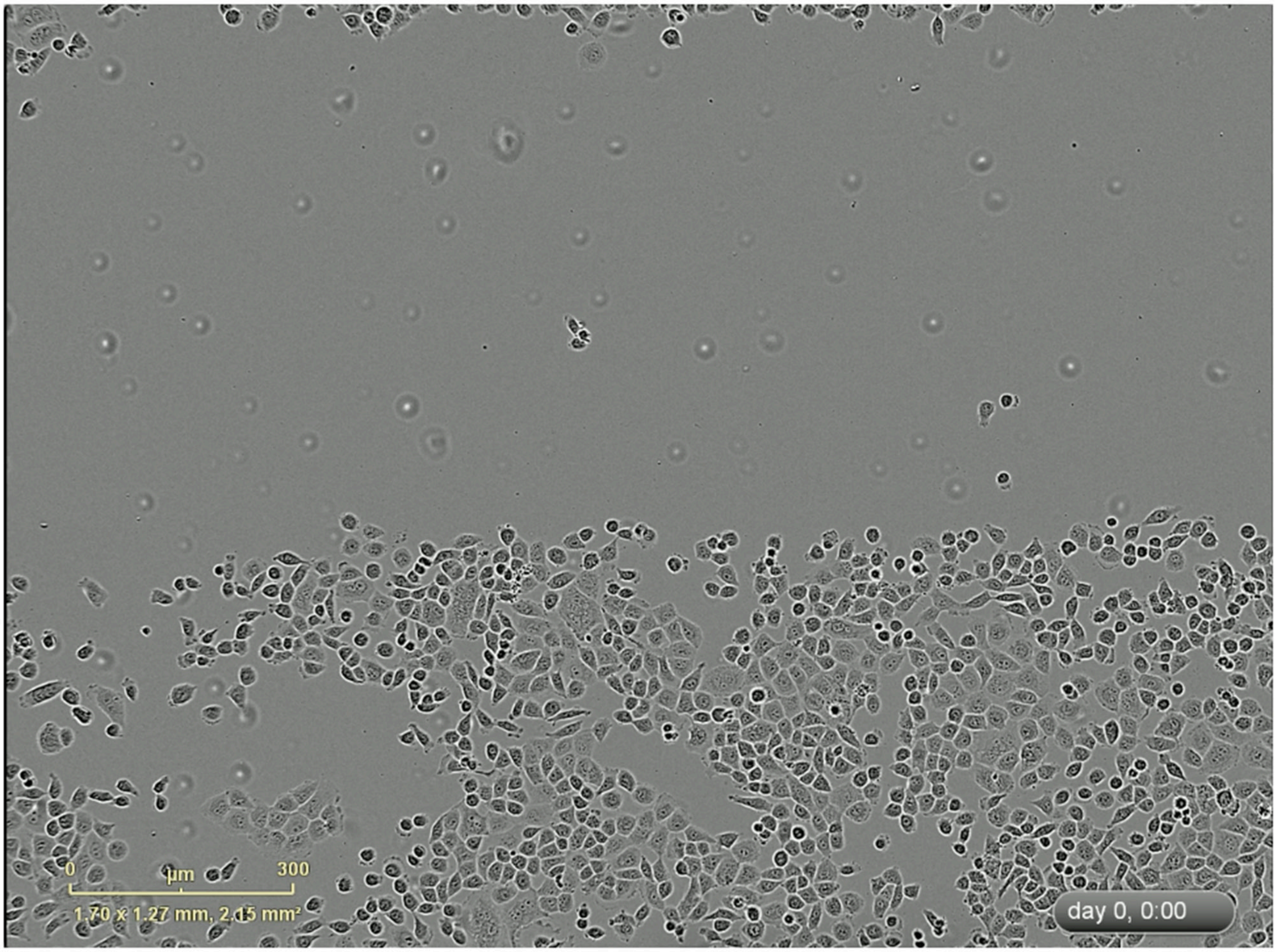


**Supplementary Video 2: Related to Figure 3G - Wound healing assay for C4-2 prostate cancer cells with no treatment.**

**See Supplementary Video 2**

**Supplementary Table 1: Real time primer sets information**

| Gene ID | Accession number | Primers sequence | Product length |
|---|---|---|---|
| TMPRSS2 | NM_001135099.1 | Fw: 3`- CGGCGCGGCAGGTCATATT- 5`<br>Rev: 3`- GCCGGATGCACCTCGTAGAC-5` | 192 bp |
| PMEPA1 | NM_020182.4 | Fw: 3`- CTGCGGGAATTTTTCTGCCC-5`<br>Rev: 3`- GTTTGCAGTCATCCGCACAT-5` | 199 bp |
| TRIM25 | NM_005082.4 | Fw: 3`- CTGCGGGAATTTTTCTGCCC-5`<br>Rev: 3`- GTTTGCAGTCATCCGCACAT-5` | 219 bp |
| KLK3 (PSA) | NM_001030047.1<br>NM_001030048.1<br>NM_001648.2 | HS02576345_m1 | 83 bP |
| β-catenin | NM_001904.3<br>NM_001098209 | HS00355049-m1 | 67 bp |
| Androgen receptor (AR) | NM_001011645.2<br>NM_000044.3 | HS00171172-m1 | 72 bp |
| Protein kinase D1 (PKD1) | NM_002742.2 | Hs00177037-m1 | 73 bp |
| RNA18S | NR_003286.2 | Hs03928990-g1 | 61 bp |
| Protein kinase D1 gene (PRKD1 )<br>P1: Proximal part of the promoter region<br>P2: Distal part of the promoter region | GENE ID:5587 | P1: Fw: 3`-GACTCGTCCGGGGACTACAG-5`<br>Rev: 3`-CGGCGACTTACCTTCTGGTC-5`<br>P2: Fw: 3`-AGAAGTGCGTAGTCCTCCAG-5`<br>Rev: 5`-AGCCGGGATAGGACCGAG-3` | 77 bP<br>221 bp |
| AR (promoter region) | GENE ID: 367 | Fw: 3`-AGGGAAAAAGGGCCGAGCTA-5`<br>Rev: 3`-TCCTCCGAGTCTTTAGCAGC- 5` | 185 bp |
| CTNNB1 Promoter region | GENE ID:1499 | Fw: 3`-AGGCGAAGGTGATGGCTTAC-5`<br>Rev: 3`-CCATTTGGCCAGCTTTGGAG-5` | 199 bp |
| PRKD1 (based on CHIP seq. data) | GENE ID:5587 | FW: GAATTTACCATTTAGGATTTTGCCC<br>Rev: ACTAAGCAGTCAGTCCATGCTA | 103bp |

**Supplementary Table 2: Position of E-box sequence on *PrKD1***

| | Starting position of E-box CTCGTG | Starting position of E-box CTCTTG |
|---|---|---|
| 1 | 29901258 | 29926230 |
| 2 | 29882881 | 29918537 |
| 3 | 29871813 | 29916561 |
| 4 | 29844304 | 29912404 |
| 5 | 29840883 | 29900486 |
| 6 | 29801393 | 29900361 |
| 7 | 29773333 | **29897492*** |
| 8 | 29772167 | 29896145 |
| 9 | 29727202 | 29894154 |
| 10 | 29704650 | 29891178 |
| 11 | 29690069 | 29877850 |
| 12 | 29673033 | 28977207 |
| 13 | 29621319 | 29876452 |
| 14 | 29596603 | 29875385 |

Gene sequence (GRCh.p7): 29576479-29927693; Numbers shows the staring position of E-box sequence. MYC/MAX complex attaches to E-box sequences on target genes. E-box sequence CTCGTG has found 14 times in the whole gene whereas E-box sequence CTCTTG has found 155 throughout the gene.” *” shows the E-box near exon 2, located in the 166bp that recruited to beta-catenin in CHIP sequencing.
